# Development of a self-report instrument for measuring online teaching practices and discussion facilitation

**DOI:** 10.1371/journal.pone.0275880

**Published:** 2022-10-07

**Authors:** Whitney DeCamp, Brian Horvitz, Regina L. Garza Mitchell, Megan Grunert Kowalske, Cherrelle Singleton

**Affiliations:** 1 Department of Sociology, Western Michigan University, Kalamazoo, Michigan, United States of America; 2 Department of Educational Leadership, Research and Technology, Western Michigan University, Kalamazoo, Michigan, United States of America; 3 Department of Chemistry and the Mallinson Institute for Science Education, Western Michigan University, Kalamazoo, Michigan, United States of America; Central China Normal University, CHINA

## Abstract

Online learning in higher education has been increasing for many years. This is happening across all of higher education and it is happening more specifically within STEM fields. The growth of online learning has significantly accelerated the past couple of years during the COVID-19 pandemic as colleges and universities have sought ways to continue educating students while also keeping students, faculty and staff safe. As result, many college faculty and instructors across all fields of study including STEM fields have made and continue to make the transition to teaching online for the first time. Teaching in an online environment is different from traditional classroom teaching in many ways and presents a unique set of challenges to college instructors. This study documents the development of an instrument used for instructors to self-report their instructional techniques and practices. Data from 251 instructors is also used to examine how this instrument can be used to better understand particular practices, with a focus in this study on discussion facilitation. The results align with the Community of Inquiry framework, including indicating that teaching through discussion forums involves direct contribution and/or facilitation.

## Introduction

Online education is a rapidly growing component of higher education, with enrollment in online courses accounting for an increasing proportion of the learning experience. In fall 2014, for example, 5.8 million students were enrolled in online classes in the United States [1]. By fall 2019, that had risen to 7.3 million [2], which is more than a 25% increase in just five years. This is, of course, prior to the COVID-19 pandemic, which necessitated increasing reliance on remote instruction and may have a lasting impact on the proportion of higher education that is available online. Whereas instructional techniques in the physical classroom have evolved into best practices over hundreds of years, the development of instructional techniques for the online classroom has thus occurred over a much shorter timetable. Teaching in an online environment is different from traditional classroom teaching in many ways and presents a unique set of challenges to college instructors. Although there exists a robust effort to study online teaching, there remains much that we do not yet fully understand and much to accomplish before time-tested and validated best practices can emerge.

The importance of effective STEM (science, technology, engineering, and mathematics) teaching has been stressed in numerous reports, including the President’s Council of Advisors on Science and Technology [3], the American Association for the Advancement of Science (AAAS) [4], and the National Research Council’s Discipline-Based Education Research Report [5]. University staff and faculty are engaged in continuing initiatives to answer these calls. These initiatives work to cultivate public scientific literacy [6], enhance workforce readiness [7], and increase the competitiveness of the United States in the global economy [3]. A central component of many change initiatives has been to encourage postsecondary instructors to adopt pedagogical approaches based in research on how people learn [4, 8].

The past decade has seen tremendous growth in online learning in higher education [9]. This growth skyrocketed in 2020 with the onset of the COVID-19 pandemic, with many institutions moving instruction online. This is illustrated by data from the National Center for Education Statistics who report that from Fall 2019 to Fall 2020, the number of students taking any distance education courses almost doubled from 7,254,455 (37%) to 14,056,994 (74%) [10]. Although there are no data on how much of this growth happened in STEM fields, there is other evidence of similar growth. In the report “Teaching Online: STEM Education in the Time of COVID” [11], 896 STEM teaching faculty spanning 49 states responded to a survey of teaching practices during the pandemic. Among the respondents, 43% reported having taught an online course prior to the pandemic and 73% reported having converted a course to an online modality since the pandemic began, which is a monumental shift. Continued research is needed to see to what degree this move online continues to shape STEM instruction in higher education.

To understand the nature of teaching practices in STEM, stakeholders need valid and reliable information about current and continuing conditions in the classroom. In response to the need for shared language in measuring postsecondary teaching practices, the AAAS, with support from the NSF, hosted a workshop that explored the state of describing and measuring undergraduate STEM education. The report produced by this workshop documents the numerous methods by which to measure postsecondary instructional practices, including faculty surveys, student surveys, interviews, class observations, and portfolio/artifact analysis [4]. These methods are predominantly content-oriented and focus on face-to-face classrooms, yet the use of technology alters instructional realities and practices, indicating the importance of investigating online STEM teaching practices. To address this need for robust instruments that measure online instructional practices in STEM coursework, we worked to create the instrument described in this study which can aid researchers and that STEM instructors can use to develop and improve as online teachers.

The present study documents the development process of an instrument for measuring online teaching approaches and techniques in undergraduate STEM courses. This involved a four-phase process and 251 participants. The final product includes measures for many elements in online instruction. To provide a more detailed understanding of one such element, this study includes an analysis of 251 responses to empirically identify underlying factors that shape instruction through student-to-student interaction in asynchronous text-based discussion forums.

### Theoretical framework

To help ensure the instrument we create reflects and encourages effective online teaching practices, we sought a conceptual framework that is well supported in the educational literature. The approach we settled on is the Community of Inquiry (CoI) framework, which views meaningful learning experiences as generated through three interdependent elements: social presence, cognitive presence, and teaching presence [12]. The CoI framework was developed to acknowledge both the cognitive and social dimensions of online learning. It has been used widely since first introduced, including two 2010 special issues of the Internet and Higher Education. CoI research has also included examinations of epistemic engagement in online learning [13], the development of community in blended learning [14], and the effects of instructional methods on the quality of student interaction [15].

Teaching presence, which is the focus of this study of online discussions, is defined as “the design, facilitation and direction of cognitive and social processes for the purpose of realizing personally meaningful and educationally worthwhile learning outcomes” [16, p. 163], and has been argued to be the “backbone of the community” in context of discussions [17, p. 159]. A large body of evidence supports the importance of teaching presence for successful online teaching [e.g., 13, 18, 19]. Teaching presence is an important factor in determining students’ satisfaction, perceived learning, and sense of community [16]. Teaching presence can further be understood through its own three interrelated elements: 1) Instructional design, which is “the planning and design of the structure, process, interaction and evaluation aspects of the online course” [16, p. 163]; 2) Facilitating discourse, which is “the means by which students are engaged in interacting about and building upon the information provided in the course instructional materials” [16, p. 164]; and 3) Direct instruction, which is “the instructor’s provision of intellectual and scholarly leadership, in part through sharing their subject matter knowledge with the students” [16, p. 164].

This model of teaching presence, situated within the larger CoI framework, focuses on how the instructor and students interact with each other and with course content. This framework is similar to several observational protocols (e.g. RTOP, TDOP), instructor surveys designed for face-to-face classrooms, and the survey developed for Henderson’s NSF- WIDER project [20]. For example, facilitating discourse is similar to the student-student interaction construct described by Walter et al. [20] or student-teacher interactions and dialogue category elicited by the TDOP [21]. Since the CoI framework is widely used within the online education research community, and aligns with the constructs developed in other work, the model fits with this project’s focus on teaching practices in online STEM environments.

### Online discussions

As explained above, discourse is one of the three interrelated elements of teaching presence within the CoI framework. Discourse among students and between students and teachers in an online learning environment can be categorized into two types of communication: asynchronous and synchronous. Asynchronous online learning, commonly facilitated by online tools such as discussion boards, relies on communication amongst and between students and teachers who may or may not be online at the same time. Synchronous online learning, commonly facilitated by online tools such as video conferencing and chats, relies on real-time communication amongst and between students and teachers who are online at the same time [22].

Studies on asynchronous learning environments have reported increased student perception of learning and satisfaction when they have more interaction with their instructors [23], when a discussion board is moderated by an instructor [24], and when instructors explicitly make efforts to motivate students in their discussions [25]. There is also a study that reported the number of instructor responses in discussion boards correlates positively with the number of student responses [26]. Conversely, there are also studies that report negative relationships between instructor and student participation in asynchronous discussions. In their study, Mazzolini and Madison categorized instructor participation in three ways: sage on the stage (high participation), guide on the side (moderate participation), and ghost in the wings (low participation) [27]. They examined their effects on students’ rate and length of posts and found instructor participation style had little correlation with student participation. In a follow-up study, Mazzolini and Madison looked at the content of instructors’ posts. They found no correlation between the type of instructor post and student posting rate or length [28]. They also found that as instructor posts increased, student posts decreased in frequency and length. An, Shin, and Lim found that when instructors participate a lot in discussion boards, students replied more to instructor comments and less to peer comments [29], and Dixson, Kuhlhorst, and Reiff reported that instructor participation led to decreased student participation [19].

There are also studies that report on how instructor participation impacts student participation. Nandi, Hamilton and Harland examined the quality of asynchronous discussions and identified themes related to instructor participation [30]. They found that instructors do play an active role in initiating discussions and moving those discussion forward, confirming instructors’ role as an important one. Arend reported that critical thinking among students is improved when instructors put a more consistent emphasis on discussion participation and when instructor facilitation is less frequent but more purposeful [31].

Although prior research provides some insights into the impact of discussions, we have little information on the prevalence of online discussions. Likewise, the few studies that have examined the type of instructor interaction in online discussions are quite dated given the advances and exponential growth in online education, leaving much unknown about the current nature of online discussions.

Based on extant literature and identified gaps in knowledge regarding the facilitation of discourse in asynchronous online discussions, the following research questions are used to guide this study:

1. What proportion of instructors of online courses use asynchronous discussion forums in their courses?;
2. What is the extent and style of instructor participation in discussions?; and
3. What are the correlates/predictors of discussion usage and participation?

The analyses that follow use the self-report data collected with our new instrument to provide new insights in these areas.

## Instrument development and methods

To develop our instrument, we used an iterative mixed methods design, which involved collecting and analyzing rich qualitative data (observations, interviews) to explore the phenomenon prior to quantitative data collection (survey development and validation). This approach is well suited to instrument development, as it places critical content analysis of the literature and descriptive observational data before and to purposefully inform instrument development [32, 33]. A similar method was used to develop a valid and reliable survey of postsecondary instructional practices for face-to-face classrooms [20, 34]. Mixed method approaches are generally considered superior to single method approaches as they can answer research questions that other methodologies cannot and thus provide stronger inferences [35].

The development of our instrument was split into four phases. Phase 1 focused on developing the set of constructs that describe online teaching. Phase 2 focused on designing an alpha version of the instrument. Phase 3 focused on testing and revising the alpha version of the instrument. Phase 4 focused on testing, revising and validating the beta version of the instrument. In light of the calls for better understanding of instructional approaches and effectiveness in STEM [3–8], all phases of this study used STEM courses and instructors as the focus. A wider scope may have resulted in an instrument more generalized but less sensitive to the instructional techniques of STEM. This study was reviewed and approved by the Western Michigan University institutional review board. All participants provided written consent.

### Phase 1

The purpose of the critical content analysis was to organize the literature on online postsecondary STEM classrooms into the CoI teaching presence elements: instructional design and organization, facilitating discourse, and direct instruction. This process produced an organized set of elements that describes teaching in online learning environments, including description of what can be observed, what can be self-reported by survey, and those we cannot measure. In this process, potential measurement target areas were based on the Teaching Presence element of the CoI framework [16] which included instructional design and organization, facilitating discourse, and direct instruction.

In addition to identifying constructs from CoI literature, we also recruited three online STEM undergraduate instructors who gave us permission to observe their completed courses and whom we interviewed. Our research team conducted rich, open-ended observations and extended semi-structured interviews. Data were analyzed iteratively using a constant comparative method [36] by the project team through extended discussions about what is occurring, how we know, and why it is important. We then mapped the results of these open-ended observations and interviews to the constructs identified from the literature. At the end of this process we identified a tentative set of constructs (and their definitions) that could be used to fully describe online instruction in the context of undergraduate STEM.

We then submitted our set of constructs to a panel of experts for expert validation. The expert panel included the four members of our project’s advisory board plus ten additional experienced online instructors recruited using a snowball technique [37] beginning with colleagues and members of our advisory panel. This panel individually reviewed the set of constructs and provided detailed feedback which the research team used to revise the set.

### Phase 2

The goal of this phase was to design a first version of the instrument. In the first step in this phase, the research team took the set of constructs produced in Phase 1 and generated items for inclusion in the instrument. This was a group process that involved discussion among the project team members. The items agreed upon by the team were organized into an alpha version of the instrument. This alpha version was then reviewed and validated by the same expert panel used in Phase 1. The panel used their knowledge and experience with online courses to provide critiques regarding the extent to which the items reflect the concepts upon which they are based and whether they have face validity. Their feedback was used to revise the alpha version of the instrument that was tested in Phase 3. This was an iterative process, with the panel assessing revisions until no further changes were deemed needed.

### Phase 3

The focus of this third phase of our project was to test, revise and validate the version of the instrument that came out of Phase 2. The instrument was piloted on three instructors of completed online undergraduate STEM courses, including conducting additional semi-structured interviews with these participants. We then came to together as a team to discuss these results and used this data to make revisions to the instrument. The revised instrument was then subjected again to review by the project’s expert panel.

### Phase 4

The final phase of the project was to collect data from instructors in multiple STEM disciplines. In order to collect data from online education instructors at multiple universities, a list of 19 public higher education institutions that regularly offered a large number of online STEM courses was compiled. This step of data collection was completed before the COVID-19 pandemic and therefore reflects institutions with a history of online teaching that predates the massive shift to online education that occurred in Spring 2020. From these 19 institutions, a sampling frame was developed by reviewing course schedules and preparing a list of instructors at each university who were teaching online STEM undergraduate courses. Schedules for Fall 2019, Winter 2019–2020 (at institutions that have Winter sessions), and Spring 2020 were each reviewed to produce as comprehensive a list as possible. In total, 1,991 unique instructors were identified across the 19 universities, and 1,500 of them were randomly selected to receive an invitation to participate in the survey. Although the sample includes Spring 2020, which was a semester partially affected by the pandemic, the schedules were reviewed in January 2020 before the shift to online occurred at any U.S. institutions and therefore reflect courses that were planned to be offered online regardless of the pandemic.

Personalized email invitations to participate in the survey were sent to each of the randomly selected instructors in January 2021. Each individual was offered a $50 gift card to Amazon, Target, or Barnes & Noble for their participation. Reminder emails were later sent to any individuals who neither completed the survey nor unsubscribed from the email list. Overall, 251 instructors representing all 19 institutions completed the survey, corresponding to a 16.7% response rate, in comparison to a target sample of 250 (the number of gift cards budgeted).

Overall, the sample was demographically diverse. For gender identity, 56.2% were male and 43.4% were female, with the remaining 0.4% (n = 1) identifying as agender. For race/ethnicity, 72.9% were non-Hispanic white; 15.5% were Asian; 6.0% were Hispanic, Latino, or Spanish origin; 3.2% were Black or African American; 1.2% were Middle Eastern or North African; 0.8% (n = 2) were American Indian / Native American; and 0.4% (n = 1) identified with multiple non-White racial/ethnic identities. For appointment status, 24.3% reported being tenured, 7.2% tenure-track, and 68.5% reported a non-tenure/track appointment.

## Measuring discussions

To measure whether an asynchronous discussion was present in the course, participants were asked the following yes/no question: “Do you assign discussion forums? For example, student-to-student discussions to further understanding of course topics.” To measure whether the instructor contributes to the discussion, the following yes/no question was asked: “Do you contribute, other than an initial prompting question, in your discussion forums for [course name]?” Finally, participants were provided a prompt to “please describe how often you contribute to discussion forums for the following reasons” and presented with a series of 12 types of contributions. Logical skip patterns were used to present these questions only when they were applicable (i.e., participants who said that they don’t use discussions were not presented with the other two prompts; participants who said they do not contribute to discussions were not presented with the final prompt). Univariate and factor analyses will be presented in the results section to explore the data from these indicators. For a reproduction of the questions used, see S1 Appendix.

### Predictors

Because the survey was designed to focus on a particular course rather than the instructor, the inclusion of variables about the instructors themselves was limited. For example, there were no questions about pedagogical training. Nevertheless, there are a number of indicators in the survey that might be informative about correlates and influences on decisions about discussion approaches. Participants were asked for the total number of students in the course, which may influence the feasibility of having discussions and the ability for the instructor to contribute. Instructors were also asked for the number of times they have taught this course online, how many tears they have been teaching, and how many years they have been teaching online. Each of these is indicative of professional experience. Demographic variables were included as control variables, including gender (reference = male), race (reference = non-Hispanic white), and appointment type (reference = tenured). The descriptive statistics for these variables is displayed in Table 1.

**Table 1:** Descriptive statistics of predictors.

Item	Mean	SD	Min	Max	n
Number of Students	79.70	75.72	4.00	300.00^†^	248
Times Taught	6.38	5.64	0.00	20.00^†^	250
Years Teaching	13.17	7.97	1.00	25.00^†^	240
Years Teaching Online	6.11	3.87	0.50	12.00^†^	242
Gender (Female)	(43.6%)		0.00	1.00	250
Race (Non-White)	(27.1%)		0.00	1.00	251
Tenure-Track Appointment	(7.17%)		0.00	1.00	251
Contingent Appointment	(68.53%)		0.00	1.00	251

^†^ Outliers were recoded to a maximum set based on the observed distribution (approximately one standard deviation above the mean) in order to prevent influential outliers in the regression models. The original maximums were 1600, 89, 49, and 33 respectively.

### Analytic strategy

Analyses began with SPSS 26.0 via a univariate examination of the variables measuring the presence and nature of discussions (RQ1). From there, factor analysis was used to further explore the types of contributions by instructors to identify common themes (RQ2). Finally, a series of logistic regressions were performed in SAS 9.4 using PROC LOGITISTIC in order to estimate the correlates/predictors of discussion usage and style. In order to retain as many cases as possible for the analysis, missing data for the predictor variables were imputed using PROC MI. Data were not imputed for any dependent variables (only one case had missing data on a dependent variable), and inapplicable cases were excluded as appropriate (e.g., instructors who do not use discussions are excluded from analyses predicting discussion contribution style).

## Results

As described above, this instrument development process employed an iterative mixed methods design. In Phase 1, we conducted a critical content analysis of CoI teaching presence elements. We also observed a set of completed online courses and conducted extended semi-structured interviews of the courses’ instructors. These qualitative data were used to develop the set of constructs that were turned into instrument items in Phase 2. Versions of this instrument were then subjected to iterative rounds of quantitative testing and revision in Phases 3 and 4. The final version of the instrument is included as S1 Appendix.

The data from the 251 instructors in Phase 4 were analyzed to better understand how they use discussions in their courses. Upon being asked whether the instructor assigns discussion forums, 64.0% (n = 160) indicated that they do and 36.0% (n = 90) indicated that they do not (one participant did not answer the question). Among those who do use discussion forums, 72.5% (n = 116) reported that they contribute to the discussion beyond providing the initial prompt and 27.5% (n = 44) reported that they do not.

The univariate analyses regarding the type of instructor contribution are displayed in Table 2 (presented in the order they were presented to participants). The most common types of contributions were guiding the class towards understanding course topics in a way to help the student clarify their thinking (#7), providing encouragement within a discussion (#5), and diagnosing misconceptions within a discussion (#4), with the majority of contributing instructors reporting these types of contributions often or always. Between one-third and one-half of contributing instructors reported using most of the remaining types of contributions often or always. Only two types of contributions were reported as being used often or always by less than one-third of contributing instructors, including summarizing a discussion (#3) and building a consensus to a discussion (#2). All twelve contribution types were reportedly used by the majority of instructors at least some of the time.

**Table 2:** Discussion involvement type.

		Frequencies (percentages)						Factor Loadings (Varimax Rotation)	
#	Item	Never	Rarely	Some-times	Often	Always		Factor 1	Factor 2
1.	Adding information to a discussion	3.4	11.2	44.0	25.9	15.5		.455	.515
2.	Building a consensus to a discussion	22.4	22.4	34.5	14.7	6.0		.433	.688
3.	Summarizing a discussion	19.8	24.1	26.7	19.0	10.3		.238	.728
4.	Diagnosing misconceptions within a discussion	3.4	12.1	31.0	32.8	20.7		.357	.617
5.	Providing encouragement within a discussion	4.3	8.6	27.6	31.9	27.6		.026	.775
6.	Identifying and clarifying areas of agreement and disagreement on course topics to help students learn	12.9	12.1	35.3	25.9	13.8		.548	.623
7.	Guiding the class towards understanding course topics in a way to help the student clarify their thinking	8.6	8.6	21.6	31.9	29.3		.590	.441
8.	Keeping students engaged and participating in productive dialogue	9.5	12.1	31.9	24.1	22.4		.811	.294
9.	Keeping students on task	15.5	20.7	19.0	27.6	17.2		.857	.124
10.	Encouraging course participants to explore new concepts associated with the course	7.8	17.2	35.3	22.4	17.2		.721	.384
11.	Reinforcing the development of a sense of community among course participants	6.0	21.6	24.1	29.3	19.0		.442	.494
12.	Focusing the discussion back to relevant issues to help student learning	11.2	24.1	29.3	24.1	11.2		.818	.239

n = 116.

A factor analysis of the responses to the twelve types of contributions is also displayed in Table 2. The analysis indicates that two factors can be extracted from these items, suggesting that there are two themes in contribution types. In order to further explore these themes, additional factor analyses and reliability analyses were estimated based on the factor loadings of the original factor analysis. Based on the factor loadings observed, variables were selected based on two inclusion criteria: factor loadings greater than .5 for the factor in question and factor loadings at least .1 greater than for the alternative factor. This approach produced two mutually-exclusive sets of contribution types.

An analysis of the first factor is displayed in Table 3. This factor included items related to: guiding students toward understanding, encouraging engagement and productive dialog, maintaining focus, student exploration of new concepts, and guiding students away from non-relevant discussion. These items indicate an emphasis on student contributions to the discussions with the instructor acting as a navigator. Based on the types of contributions included in this factor, it has been identified as “facilitation.” An analysis of the second factor is displayed in Table 4. This factor included items related to: consensus building, summarizing, diagnosing misconceptions, and providing encouragement. These items indicate an emphasis on instructor contributions to the discussions with the instructor injecting substantive contributions with a more hands-on approach. The item related to providing encouragement (which also has the lowest factor loading) is less overtly about adding substantive information than the others, and more information would be useful in better understanding its relation to the others. Based on the types of contributions included in this factor, it has been identified as “direct contribution.” Together, these represent two different approaches an instructor might take with discussions; one that focuses on classroom management in order to keep discussions productive, and another that focuses on a more hands-on approach for interaction. Although these approaches are different, they are not necessarily mutually exclusive. To the contrary, the two factors have a positive correlation (r = .646, n = 116, p < .01), suggesting that an instructor who uses one of these approaches has an increased likelihood of also using the other. Overall, 33.6% of contributing instructors are above the mean on both factors, 16.4% are above the mean for only the facilitation factor, 15.5% are above the mean for only the direct contribution factor, and the remaining 34.5% are below the mean for both factors, suggesting a wide variety of approaches that mix and match different styles.

**Table 3:** Factor 1 –facilitation.

#	Item	Factor Loading
7.	Guiding the class towards understanding course topics in a way to help the student clarify their thinking	.748
8.	Keeping students engaged and participating in productive dialogue	.869
9.	Keeping students on task	.841
10.	Encouraging course participants to explore new concepts associated with the course	.828
12.	Focusing the discussion back to relevant issues to help student learning	.827

n = 116; α = .880; Eigenvalues = 3.392.

**Table 4:** Factor 2 –direct contribution.

#	Item	Factor Loading
2.	Building a consensus to a discussion	.867
3.	Summarizing a discussion	.844
4.	Diagnosing misconceptions within a discussion	.717
5.	Providing encouragement within a discussion	.642

n = 116; α = .773; Eigenvalues = 2.390.

The logistic regression results predicting whether a discussion is used are presented in Table 5 (Model 1). Each increase in the number of times an instructor has taught a course is associated with a significant increase in the probability that a discussion is currently included in the course (β = .260, OR = 1.087, p < .05). No other predictors have significant effects in this model. It is important to keep in mind that whether a discussion is offered is a pedagogical decision and is likely influenced by variables not included here, so this is not designed or expected to be a comprehensive model of all relevant predictors. The logistic regression results predicting whether the instructor contributes to a discussion are also presented in Table 5 (Model 2). Just as with the previous model, the number of times they have taught a course is the only significant predictor, though the direction is reversed here. Each increase in the number of times an instructor has taught a course is associated with a significant decrease in the probability that the instructor contributes to the discussion (β = -.337, OR = .904, p < .01).

**Table 5:** Logistic regression predicting discussions and contributions.

	Model 1: Any Discussion						Model 2: Contributes					
Predictor	b	SE	β	OR	p		b	SE	β	OR	p	
Number of Students	-0.002	0.002	-0.085	0.998	0.264		0.001	0.003	0.055	1.001	0.603	
Times Taught	0.084	0.033	0.260	1.087	0.011	*	-0.101	0.039	-0.337	0.904	0.009	**
Years Teaching	0.007	0.023	0.032	1.007	0.750		0.001	0.033	0.006	1.001	0.967	
Years Teaching Online	-0.008	0.051	-0.016	0.993	0.883		0.077	0.078	0.161	1.079	0.324	
Gender (Female)	0.349	0.285	0.095	1.417	0.221		-0.270	0.387	-0.074	0.763	0.485	
Race (Non-White)	-0.178	0.309	-0.044	0.837	0.563		0.942	0.524	0.224	2.566	0.072	
Tenure-Track Appointment	0.293	0.616	0.042	1.341	0.634		-0.711	0.932	-0.100	0.491	0.445	
Contingent Appointment	0.214	0.362	0.055	1.238	0.555		-0.740	0.568	-0.188	0.477	0.193	
Sample Size (n)	250						160					
Nagelkerke R^2^	.070						.127					

* p < .05, two-tailed

** p < .01, two-tailed; b = unstandardized coefficient; SE = standard error of b; β = standardized coefficient; OR = odds ratio.

The logistic regression results predicting whether a contributing instructor is above the mean for the facilitation contribution factor are presented in Table 6 (Model 3). None of the experience-related predictors are significant, though instructors who identify as a racial/ethnic minority are significantly more likely to be above the mean for facilitation-type contributions than are non-Hispanic white instructors (β = .236, OR = 2.573, p < .05). The logistic regression results predicting whether a contributing instructor is above the mean for the direct contribution factor are presented in Table 6 (Model 4), though none of the predictors have a significant effect. Together, these suggest that the influences that lead to the type of contributions instructors make are generally not related to the number of students or the length of the instructor’s experience.

**Table 6:** Logistic regression predicting discussion contribution type.

	Model 3: Facilitation						Model 4: Direct Contribution				
Predictor	b	SE	β	OR	p		b	SE	β	OR	p
Number of Students	-0.002	0.003	-0.081	0.998	0.482		0.002	0.003	0.072	1.002	0.510
Times Taught	-0.043	0.042	-0.137	0.958	0.305		-0.038	0.041	-0.120	0.963	0.351
Years Teaching	-0.027	0.034	-0.117	0.974	0.424		-0.028	0.032	-0.119	0.973	0.393
Years Teaching Online	0.115	0.076	0.243	1.122	0.132		0.092	0.072	0.194	1.096	0.206
Gender (Female)	0.340	0.409	0.093	1.405	0.406		-0.015	0.398	-0.004	0.986	0.971
Race (Non-White)	0.945	0.478	0.236	2.573	0.048	*	0.360	0.453	0.090	1.433	0.428
Tenure-Track Appointment	-0.602	0.943	-0.085	0.548	0.523		-0.633	0.932	-0.089	0.531	0.497
Contingent Appointment	-0.760	0.528	-0.198	0.468	0.150		-0.138	0.505	-0.036	0.871	0.785
Sample Size (n)	116						116				
Nagelkerke R^2^	.113						.037				

* p < .05, two-tailed

** p < .01, two-tailed; b = unstandardized coefficient; SE = standard error of b; β = standardized coefficient; OR = odds ratio.

## Discussion

The CoI framework has been used to explain what constitutes a meaningful learning environment in online courses. CoI predicts that teaching presence, one of the necessities for effective learning, includes three components: instructional design, facilitating discourse, and direct instruction [16]. Online asynchronous discussion is a tool used by instructors that can contribute to teaching presence, and research indicates that such discussion provides various benefits in online courses [23–25]. Despite teaching theory arguing and research study evidence showing that discussions are a vital component of online learning, much is unknown about the current use of discussions in online courses.

The present study examined self-reported instructional actions for asynchronous online discussions in fully online higher education courses. The resulting analyses indicated that nearly two-thirds (64.0%) of online instructors use asynchronous discussion forums as part of their online courses. Moreover, 72.5% of those instructors (46.4% of instructors overall) participate in those discussions. This suggests that asynchronous discussions are a commonly used tool for online education and facilitate both student-student and student-instructor discourse. The CoI framework’s concept of facilitating discourse suggests that either of these types of discourse contribute to teaching presence.

The factor analysis of the data identified two underlying factors: facilitation and direct instruction. This is consistent with the CoI framework which predicts both of these elements as part of teaching presence [16]. Although the CoI framework’s other teaching presence element, instructional design, was not identified as well, it is important to note that the instructional design is what sets the stage for the other two elements, and therefore is distinct from what happens during the discussion. Thus, the results are compatible with the CoI framework’s concept of teaching presence in asynchronous discussions.

The analyses also examined the prevalence of discussions and associated instructional techniques. Although facilitation and direct instruction were identified as part of teaching presence in the CoI framework over two decades ago [12], we could identify no existing research that has attempted to use instructor characteristics to predict how these manifest in discussions (or generally). Given the dearth of information on this subject, the models estimated in this study were exploratory in nature. The analyses suggest that the more often an instructor taught the course, the more likely they were to include a discussion component, but the less likely they were to contribute to the discussion. Although this was a statistically significant finding in our analyses, we were unable to identify prior research or a theoretical explanation for this relationship, so replication and further exploration is necessary to confirm and support this finding. Expanding our knowledge of the correlates of instructional practices may be useful in better understanding the practices themselves, as well as to develop more effective strategies for encouraging instructor development and improvement. A more expansive dataset will be necessary to determine the nature of the relationships identified here. For example, these effects could be the result of additional experience, but they may also be the result of generational differences in instructional approach, or a self-selection bias in who teaches the course more often. Likewise, it could be the result of disciplinary differences. These are beyond the scope of the present study, but the results here provide a potential foundation upon which to further explore this curious relationship.

One key limitation in this study is the use of self-report data. The instrument relies on instructors to accurately report how they interact with students in discussions. The confidential, no-risk design of the data collection used makes it unlikely that instructors would feel pressured to intentionally present false responses, but other forms of human error are possible. Prior research has shown that self-report and observational data have reasonable reliability with each other [38], suggesting that instructor self-report data can be reasonably trusted, though replicating the present study with observational data would undoubtedly provide a useful confirmation. Another limitation is the focus on asynchronous discussions over synchronous discussions. At the time this project was first planned in 2014 and funded by the National Science Foundation in 2017, there was no option at our university for instructors and faculty to offer fully online courses with scheduled, synchronous meeting times. All online courses were assumed to be primarily or entirely asynchronous. This influenced our decision to focus our project primarily on asynchronous online learning. Since the Fall 2020 semester our institution has created new designations for courses that include scheduled synchronous sessions. This was directly in response to the COVID pandemic which caused the temporary halt of all face-to-face instruction in March of 2020. We acknowledge that there has been great growth in the amount of online undergraduate education being offered synchronously using tools like Zoom, WebEx and Google Meet between the start of this project and the present, which is undoubtedly a limitation of this study. Future research should build on this work by incorporating synchronous teaching approaches to the instrument and analyses.

Given that nearly two-third of instructors report using asynchronous discussions according to the results of this study, the importance of better understanding the associated instructional techniques is clear. As we work towards identifying evidence-based best practices in online STEM education, using instruments like the one developed here will help in breaking teaching presence down to its components and better understanding what effect they might have on student outcomes. Although the present study used undergraduate STEM courses as the developmental baseline for instruments based on calls to better understand STEM instructional practices [3–8], it is plausible that this self-report survey can be used or adapted for use in non-STEM areas as well, subject to further testing of this instrument.

Future research also ought to examine how this self-report survey (or some variation on it) can be used by online instructors to help them self-evaluate and improve their teaching methods, particularly as they relate to the CoI concept of teaching presence. As McCombs put it, “Teachers need self-assessment and reflection tools to help them assess fundamental beliefs and assumptions about learning, learners and teaching…” [39, p. 1] A search of the literature found no other self-report surveys for teachers’ online learning practices, particularly drawing on the CoI framework.

Although this instrument was partially used in a survey approach in the present study for development and testing, the instrument is designed to be used on an individual-level basis for self-assessment and reflection. The intent is that it can be used by individual instructors seeking better understanding of their own instructional techniques and for professional growth. This might be further facilitated by discussing a self-assessment with a colleague, which our participants who were interviewed found helpful. The self-report instrument used in this study is now available for instructors and researchers [40] under an open license (CC BY-NC-SA 4.0). Research on how this instrument can be used to help instructors reflect and improve their online teaching would be a welcome to contribution to the areas of teacher training and teacher development.

## Supporting information

S1 Appendix Questionnaire. A reduced questionnaire that includes only the questions used for the present study, as they were phrased in the January 25, 2021 version used for data collection. For the full, final questionnaire, visit: https://scholarworks.wmich.edu/instruments_teaching/.
(PDF)

S1 Data Minimal dataset. The data required to replicate all study findings reported in the study.
(SAV)
